# Evaluation of Proliferative Activity of Hawaiian Plants on PC-12 and Neuro-2a Cells and Their Effect on the TPH and TH Genes

**DOI:** 10.3390/ph18091403

**Published:** 2025-09-18

**Authors:** Pornphimon Meesakul, Tyler Shea, Xiaohua Wu, Yutaka Kuroki, Aya Wada, Shugeng Cao

**Affiliations:** 1 Department of Pharmaceutical Sciences, Daniel K. Inouye College of Pharmacy, University of Hawai’i at Hilo, 200 W. Kawili St., Hilo, HI 96720, USA; pmeesak@hawaii.edu (P.M.); xiaohua3@hawaii.edu (X.W.); 2 Chemistry Department, University of Hawai’i at Hilo, 200 W. Kawili St., Hilo, HI 96720, USA; tylerms3@hawaii.edu; 3 Delightex Pte. Ltd., 230 Victoria Street, #15-01/08 Bugis Junction Towers, Singapore 188024, Singapore; yutaka@delightexplorers.com (Y.K.); aya@delightexplorers.com (A.W.)

**Keywords:** Hawaiian plants, toxicity, neuro-2A, PC-12, gene expression, PCR

## Abstract

**Background/Objectives**: Neurotransmitters such as dopamine and serotonin are critical regulators of mood, cognition, and neuronal homeostasis. This study aimed to evaluate the neuropharmacological potential of Hawaiian plants by investigating their ability to modulate the expression of tyrosine hydroxylase (TH) and tryptophan hydroxylase (TPH), key enzymes in neurotransmitter biosynthesis. **Methods**: A total of 108 aqueous and methanolic extracts of Hawaiian plants were screened for cytotoxicity against PC-12 and Neuro-2A cells using the MTT assay. Fifty-six non-toxic extracts were selected and further analyzed for TH and TPH expression via quantitative real-time PCR (qPCR). **Results**: Several extracts significantly upregulated TH and TPH expression without inducing cytotoxicity. Extracts derived from *Morinda citrifolia*, *Pipturus albidus*, and *Hedychium coronarium* showed the most notable activity, suggesting their potential to enhance dopaminergic and serotonergic pathways. **Conclusions**: The findings highlight the promise of native Hawaiian flora as sources of neuroactive compounds that may support neuroprotection and regeneration. These results provide a foundation for in vivo studies and further exploration of novel neurotherapeutic agents.

## 1. Introduction

Neurotransmitters are essential chemicals involved in the transmission of signals across synapses in the nervous system [1,2]. Among these, dopamine and serotonin are two of the most studied due to their involvement in motor control, mood regulation, learning, memory, and reward pathways [3,4,5]. Tyrosine hydroxylase (TH) is the rate-limiting enzyme in the biosynthesis of catecholamines such as dopamine [6,7], while Tryptophan hydroxylase (TPH) plays a similar role in the production of serotonin [8,9,10]. Dysregulation in the expression or function of these enzymes can result in neurological and psychiatric disorders such as Parkinson’s disease, depression, and schizophrenia [1,3,4,10,11].

Plant-based compounds have emerged as promising candidates in the treatment and management of these disorders due to their wide variety of bioactive constituents and typically lower toxicity profiles compared to synthetic drugs. Hawaiian plants are of particular interest given their unique phytochemical diversity, stemming from the islands’ isolated evolution and rich ethnomedicinal history [12,13,14,15]. Investigating their biological activity not only supports drug discovery efforts but also contributes to the conservation and sustainable use of local biodiversity [14,15].

Previous research has demonstrated neuroprotective effects of various tropical and endemic plants through antioxidant, anti-inflammatory, and neurogenesis-enhancing mechanisms [14,15,16,17]. For instance, *Morinda citrifolia* (Noni) has shown promise in improving cognitive function and reducing neuronal damage [16], while *Hedychium coronarium* extracts have demonstrated significant antioxidant activities [17].

This study integrates cytotoxicity assays and gene expression analysis to evaluate the neurological potential of aqueous and methanolic extracts derived from Hawaiian plants. Focusing on PC-12 and Neuro-2A cells as in vitro neuronal models, we aim to identify plant extracts that are non-toxic and capable of modulating TH and TPH gene expression, thereby uncovering their potential roles in dopaminergic and serotonergic signaling.

## 2. Results

### 2.1. The Toxicity of PC-12 and Neuro-2A Cell Lines

**Figure 1:**
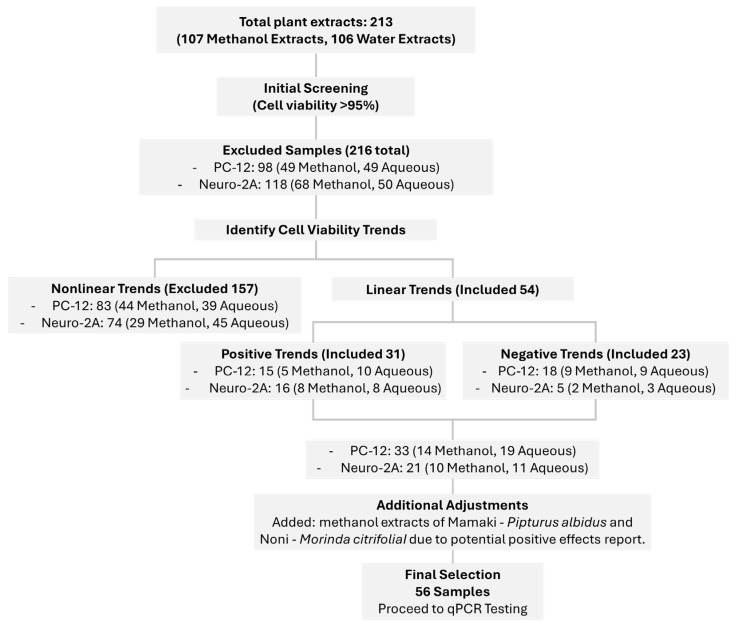
The selection process and final samples chosen for further qPCR testing.

Traditionally, plant materials are prepared by steeping in hot water prior to consumption. While this method aligns with cultural practices, it is limited in its ability to extract non-polar bioactive compounds. To better represent both traditional use and maximize phytochemical diversity, we employed both aqueous and methanolic extraction methods. A total of 108 plant extracts, including both methanol and aqueous extracts, were evaluated using the MTT assay on PC-12 and Neuro-2A cell lines at concentrations of 10, 50, and 100 µg/mL, with each concentration tested in triplicate. The initial toxicity screening results are presented in Figures S1–S4, showing two categories of cell viability trends (linear and nonlinear). The linear trends were further classified into two types: positive and negative. Based on these results, we selected 56 samples (Tables S1 and S2) for further qPCR testing, as they demonstrated cell viability greater than 95% in both cell types at all three concentrations. Nonlinear trends of cell viability were excluded from this selection. Additionally, we included methanol extracts of *Pipturus albidus* (Mamaki) and *M. citrifolia* (Noni) due to their potential positive effects demonstrated in other studies. Notably, *P. albidus* is endemic to Hawaii, while *M. citrifolia* is native to the region and widely used in traditional medicine. The selection process and final samples chosen for further qPCR testing have been summarized as shown in Figure 1.

### 2.2. Cell Treatment, RNA Extraction, and cDNA Synthesis

A total of 56 plant extracts has been selected for qPCR testing, including 21 samples (11 aqueous and 10 methanolic extracts) tested on neuro-2A cells and 35 samples (19 aqueous and 16 methanolic extracts) tested on PC-12 cells. Both cell lines were treated with selected plant extracts non-toxic concentration at 50 µg/mL for 48 h, with controls receiving 0.1% DMSO. Following treatment, total RNA was extracted, yielding high-quality RNA with A260/A280 ratio ranging from 1.8 to 2.0 and an average concentration of approximately 300 ng/μL. cDNA was synthesized using 2 µg of RNA per reaction and subsequently used for qPCR analysis.

### 2.3. qPCR Analysis

**Figure 2:**
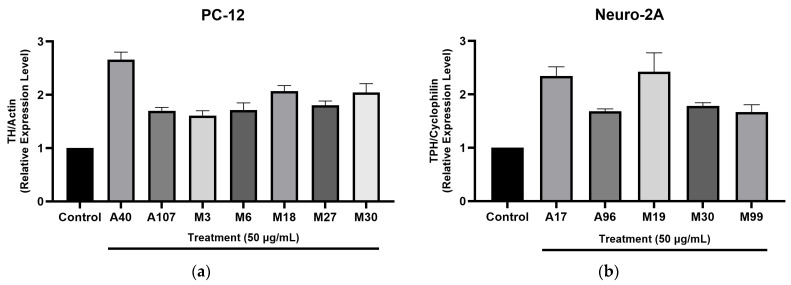
The initial PCR of the selected plants showed positive results. Total RNA was extracted cells treated as indicated. The relative expression level was determined by real-time qPCR. The data in the figure represents the mean ± SEM of triplicate experiments. *p* < 0.05 compared with the control group. (**a**) The initial PCR of the selected plants on PC-12; (**b**) The initial PCR of the selected plants on Neuro-2A.

Preliminary observations from the initial PCR runs indicate that certain plant extracts exhibited amplification (Tables S3 and S4, Figure 2). Reactions were performed using SYBR Green Master Mix and gene-specific primers (TH for PC-12, TPH for Neuro-2A; actin/cyclophilin as reference genes). Of the tested extracts, nineteen showed weak induction of target gene expression, with 2^−ΔΔCt^ values ranging from 0.21 to 0.82. In contrast, nine other extracts demonstrated moderate upregulation, with 2^−ΔΔCt^ values between 1.06 and 1.48. Notably, specific extracts such as A40 (*Coprosma ernodeoides*), A107 (*Hippobroma longiflora*), M3 (*Verbascum thapsus*), M6 (*Curcuma longa*), M18 (*C. caesia*), M27 (*Dicranopteris linearis*), and M30 (*M. citrifolia*) were able to significantly enhance the expression of TH when tested on PC-12 cells (Figure 2a) demonstrating their potential as effective modulators of TH in neurobiological contexts (Table S3).

Furthermore, qPCR analysis revealed that neuro-2A cells treated with extracts A17 (*Hedychium coronarium*), A96 (*Chenopodium oahuense*), M19 (*P. albidus*), M30 (*M. citrifolia*), and M99 (*Wikstroemia uva-ursi*) exhibited a significant elevation in TPH expression (Figure 2b). This suggests that these extracts may play a crucial role in the biosynthesis of serotonin, which is vital for various neurological functions. Additionally, among the remaining eleven plant extracts evaluated, weak expression ratios were noted, with 2^−ΔΔCt^ values ranging from 0.09 to 0.76. Conversely, five of the extracts yielded moderate expression results, with 2^−ΔΔCt^ values between 1.04 and 1.30 (Table S4). This comprehensive analysis underscores the varying efficacy of different plant extracts in influencing gene expression related to neurochemical pathways.

These results highlight specific plant extracts that are promising candidates for further study. A17 (*H. coronarium*) and M19 (*P. albidus*) show potential for neuro-2A, while A40 (*C. ernodeoides*), M18 (*C. caesia*), and M30 (*M. citrifolia*) demonstrate potential neuromodulator effects for PC-12. Further investigation into their mechanisms is warranted.

## 3. Discussion

It is important to note that this is an in vitro study, and the bioavailability, metabolic stability, and potential inactivation of these extracts in an in vivo system remain unknown and are a critical area for future research. The value of this work lies in the prioritization of specific Hawaiian plants for subsequent in-depth pharmacological and pharmacokinetic investigations.

This study employed a comprehensive cytotoxicity screening approach using the MTT assay to evaluate the effects of 108 plant extracts on two neuronal cell lines, PC-12 and Neuro-2A. This dual-cell line strategy allowed for a broader understanding of neurocompatibility across different neuronal models, which is essential given the varying responses of cell types to phytochemicals. Extracts were tested at three concentrations (10, 50, and 100 µg/mL), enabling the evaluation of dose-dependent responses including both aqueous and methanolic extracts, we ensured a wide dynamic range of response, mimicking potential dose-dependent effects observed in vivo or in traditional use.

Two primary response trends were observed in cell viability profiles, linear and nonlinear. Linear responses exhibited predictable dose-dependent changes, which were further categorized into positive (increasing viability with concentration) and negative (decreasing viability with concentration) patterns. Extracts exhibiting a positive linear trend, where cell viability increased with concentration, may contain cytoprotective or growth-stimulating compounds. Some phytochemicals, such as flavonoids and phenolic acids, exhibit antioxidant properties that mitigate oxidative damage and enhance neuronal survival [18]. Additionally, certain plant metabolites may activate cellular stress response pathways (e.g., Nrf2-mediated antioxidant defenses) that promote cell resilience [19]. The selection of extracts with greater than 95% viability for further qPCR analysis aligns with the goal of identifying non-toxic, potentially neuroprotective candidates.

Conversely, the negative linear trend, where cell viability decreased with increasing extract concentration, suggests the presence of cytotoxic compounds that impair cellular function in a dose-dependent manner. This phenomenon is commonly observed with plant-derived bioactive molecules, such as alkaloids, saponins, and certain polyphenols, which can induce oxidative stress, disrupt mitochondrial function, or trigger apoptosis at higher concentrations [20]. The methanol extracts, in particular, may contain higher levels of non-polar cytotoxic compounds that were not efficiently extracted in aqueous preparations. The consistency of this trend across triplicate experiments supports the reliability of the observed toxicity profile.

Nonlinear responses, which may indicate hormetic effects, biphasic dose responses, or complex compound-cell interactions, were excluded from downstream analysis. The nonlinear (biphasic or U-shaped) trends suggest complex interactions between phytochemicals and cellular pathways. At low concentrations, some compounds may induce mild stress that stimulates adaptive responses (hormesis), enhancing cell survival [21]. However, at higher doses, the same compounds may become toxic due to saturation of detoxification mechanisms or overwhelming pro-apoptotic signals [22]. While such extracts were excluded from qPCR analysis due to their unpredictable behavior, further mechanistic studies could elucidate their dual effects. This exclusion was deliberate, as nonlinear trends can complicate interpretation of gene expression outcomes due to their unpredictable biological behavior and may not be ideal candidates for development in the absence of detailed mechanistic studies.

From this initial screen, 56 extracts were selected based on their ability to maintain high cell viability (>95%) across all tested concentrations and in both cell lines. This high threshold was chosen to prioritize compounds with strong safety profiles, reducing the risk of cytotoxicity-related confounding effects in subsequent qPCR analysis. Importantly, the consistent viability across different concentrations suggests that these extracts are biologically inert or potentially supportive of neuronal health, warranting further molecular investigation.

In addition to viability-based selection, two plant extracts *P. albidus* (Mamaki) and *M. citrifolia* (Noni) were included due to their ethnobotanical significance and previously reported health benefits, particularly in the context of antioxidant and neuroprotective activities. Although these extracts did not strictly meet the >95% viability cut-off in all cases, their inclusion reflects a translational research approach that values traditional knowledge alongside empirical evidence. *M. citrifolia*, commonly known as Noni, has a long history of use in Polynesian medicine and has been reported to exert anti-inflammatory, antioxidant, and neuroprotective effects [14,16,23,24,25]. *P. albidus* (Mamaki), endemic to Hawaii, has gained popularity as an herbal tea and is traditionally used for fatigue, digestion, and circulatory health, though its neurological impact remains underexplored in the scientific literature [14,26,27,28,29].

The use of native and endemic Hawaiian species also aligns with growing global interest in the conservation and biomedical exploration of indigenous flora. By integrating these plants into a scientifically rigorous pipeline, our study not only contributes to neuropharmacological research but also supports the sustainable valorization of local biodiversity.

Overall, this selection process serves as a critical filtering step before gene expression profiling, helping to ensure that subsequent qPCR analysis focuses on biologically relevant and non-toxic candidates. The emphasis on extracts with consistent viability and ethnopharmacological relevance strengthens the translational potential of this research, paving the way for the identification of novel neuroprotective or neuromodulatory agents derived from natural products.

Our qPCR analysis revealed that several Hawaiian plant extracts, particularly those derived from methanol extraction, significantly modulated genes involved in neurotransmitter biosynthesis. Notably, the upregulation of TH and TPH rate-limiting enzymes in the synthesis of dopamine and serotonin, respectively, suggests that these extracts may enhance monoaminergic activity. This aligns with potential mechanisms of neuroprotection, mood regulation, and neural resilience. The greater activity observed in methanol extracts implies that mid- to non-polar phytochemical constituents such as flavonoids, iridoids, and terpenoids may underlie these effects. These findings reinforce the importance of extraction methods in the bioactivity profiling of medicinal plants.

The observed gene modulation complements earlier reports on the neuroprotective properties of *Hedychium* spp. and *M. citrifolia* (Noni), which have been attributed to their antioxidant and anti-inflammatory actions [14,16,23,24,25]. In our study, *M. citrifolia* methanol extract upregulated both TH and TPH, supporting its traditional use in mood enhancement and cognitive support. Similarly, *P. albidus* (Mamaki), a culturally significant Hawaiian herb used to relieve fatigue and anxiety, showed notable upregulation of TPH, indicating potential serotonergic activity. This molecular evidence supports the long-standing ethnobotanical use of these plants for emotional and neurological well-being.

In line with previous phytochemical research [30,31,32,33,34], the gene-modulating effects observed may be attributed to secondary metabolites such as flavonoids, iridoids, and sesquiterpenes, many of which are known to influence intracellular signaling cascades. Future studies should focus on isolating and characterizing these compounds, and on elucidating their roles in pathways regulating TH and TPH expressions, such as CREB and MAPK signaling.

This study also underscores the value of integrating traditional knowledge with contemporary molecular biology. The inclusion of endemic and native Hawaiian plants, selected not only for their empirical safety but also for their cultural relevance, strengthens the translational impact of our findings. The convergence between traditional uses, for stress relief, mood enhancement, and vitality, and the molecular data observed in this study validates the ethnopharmacological wisdom passed through generations.

Taken together, the combination of cell-based assays and qPCR profiling provides a robust platform for identifying plant extracts with potential neuromodulatory properties. These findings lay the groundwork for further mechanistic studies and may promote the development of botanical therapeutics targeting mood disorders, neuroinflammation, or neurodegenerative diseases.

## 4. Materials and Methods

### 4.1. Materials

TRIzol reagent was obtained from Sigma (St. Louis, MO, USA). β-Actin, cyclophilin, tyrosine hydroxylase (TH) and tryptophan hydroxylase (TPH) were purchased from Thermo Fisher (Waltham, MA, USA). iTag universal SYBR green supermix and iScript cDNA synthesis kit were obtained from Bio-Rad (Hercules, CA, USA). RPMI 1640 medium (ATCC^®^ 30-2001) or Dulbecco’s Modified Eagle Medium (DMEM) were bought from the American Type Culture Collection (ATCC, Manassas, VA, USA). Fetal bovine serum (FBS) was purchased from Invitrogen (Waltham, MA, USA). RNeasy Mini Kit was bought from Qiagen (Hilden, Germany).

### 4.2. Chemicals

Methanol extracts were dissolved in dimethyl sulfoxide (DMSO) while aqueous extracts were prepared in distilled water at concentration of 10 mg/mL as a stock solution, stored at −20 °C, and diluted to test concentrations with culture medium immediately prior to the experiment. The final concentration of DMSO in the culture medium was less than 0.2%. DMSO, 3-(4,5-dimethylthiazol-2-yl)-2,5-diphenyltetrazolium bromide (MTT), were purchased from Sigma (St Louis, MO, USA). Penicillin, streptomycin and 4′,6-diamidino-2-phenylindole (DAPI) were purchased from Thermo Fisher (Waltham, MA, USA).

### 4.3. Preparation of Sample

108 plants were collected from various locations on the Big Island and Honolulu, Hawaii (Endemic, native, indigenous, and naturalized plants). Collected plants were dried, blended, and extracted with methanol and water, respectively. Methanol (107 extracts) and aqueous (106 extracts) plant extracts were prepared in DMSO (for methanol extracts) and Water (for aqueous extracts) at a concentration of 10 mg/mL as a stock solution. All prepared samples were used in the treatment experiments.

### 4.4. Cell Lines and Cell Culture

PC-12 (ATCC^®^ CRL-1721, Lot: 70049614) and Neuro-2A (ATCC^®^ CCL-131 Lot: 70057026) cells were obtained from the American Type Culture Collection (ATCC, Manassas, VA, USA). Both cell lines were cultured at 37 °C in a humidified incubator with 5% CO_2_. Cells were given fresh media every three days to ensure viability. Aseptic technique was used throughout the culture process. Experiments were performed on cells between passages 2 and 14. PC-12 cells were maintained in either RPMI 1640 medium (ATCC^®^ 30-2001) or Dulbecco’s Modified Eagle Medium (DMEM) supplemented with 10% horse serum (ATCC^®^ 30-2020), 5% Fetal Bovine Serum (FBS), and penicillin-streptomycin solution (1%). Neuro-2A cells were maintained in Eagle’s Minimum Essential Medium (EMEM) containing penicillin-streptomycin solution (1%) and 10% (*v*/*v*) Fetal Bovine Serum (FBS) to create a complete growth medium.

### 4.5. Cell Viability

The MTT assay was performed to estimate cell viability following the standard protocol [35,36,37]. Cells were cultured in 75 mL vented culture flasks that were pretreated to enhance cell adhesion. PC-12 and Neuro-2A cells were each maintained in their respective media with appropriate supplements under controlled temperature, CO_2_, and humidity conditions. Following automated counting to determine cell density, the cell stock was diluted to achieve the desired seeding density 2 × 10^5^ cells per well for PC-12 and 1.8 × 10^4^ cells per well for Neuro-2A. A volume of 180 µL of the diluted cell suspension was added to each well of a 96-well plate. After seeding, the cells were allowed approximately 24 h to adhere. Each well was then treated with a single 20 µL dose of crude extract, with three replicate wells per sample. After 48 h of incubation, 10 µg of MTT reagent was added to each well, and the plates were incubated for an additional 4 h to allow the reduction of the tetrazolium salt. Cell plates were centrifuged at 1500 rpm for 15 min to pellet the cells and facilitate the removal of media. Following media removal, 100 µL of DMSO was added to each well to dissolve the formazan crystals formed from the MTT reagent. The plates were then shaken for 20 min to aid in dissolution, and absorbance was measured at 570 nm using a microplate reader. I. The percentage cell viability was calculated by taking the average absorbance value of the test wells divided by the average for the control well (which just received water or DMSO + PBS). The values for the media wells were subtracted from both values as the “blank”.

### 4.6. Cell Treatment

PC-12 and Neuro-2A cells were seeded in Petri dishes and allowed to adhere overnight (18–24 h). PC-12 cells were plated at a density of approximately 4 × 10^5^ cells/dish, while Neuro-2A cells were seeded at 2 × 10^5^ cells/dish. After the adherence period, cells were treated with selected plant extracts at a final concentration of 50 µg/mL, corresponding to their respective non-toxic concentrations, for a total duration of 48 h. Control groups received vehicle treatment (0.1% DMSO in culture medium) and were always taken from the same initial batch of cells to ensure consistency and reliability of the control values. After 48 h, treated cells were harvested. Some samples were processed immediately for further analysis, while others were stored frozen post-treatment for batch preparation [35].

### 4.7. RNA Extraction

Total RNA was extracted from the treated PC-12 and Neuro-2A cells using the RNeasy Kit. Initially, cells were pelleted by centrifugation at 3000 rpm for 10 min. The supernatant was removed, and genomic material was extracted using a standard TRI-zol protocol, followed by chloroform extraction and precipitation with 70% ethanol. The RNeasy column-based system was then used to purify RNA from the lysate. To eliminate genomic DNA contamination, the Ambion DNA-free kit (Thermo Fisher Scientific, Waltham, MA, USA), which contains DNase enzymes, was employed. The RNA samples were incubated with DNase at 37 °C for 30 min, followed by inactivation of the enzyme using the manufacturer-supplied reagent. Purified RNA was quantified using a spectrophotometer, and samples with A_260_/A_280_ ratios between 1.8 and 2.0 were selected for downstream applications [35].

### 4.8. cDNA Synthesis

To prepare RNA samples for quantitative PCR (qPCR), complementary DNA (cDNA) was synthesized using the iScript cDNA Synthesis Kit (Bio-Rad). RNA samples were quantified and normalized to a concentration of 2 µg per reaction. The cDNA synthesis protocol was performed according to the manufacturer’s instructions as follows, priming for 5 min at 25 °C then reverse transcription for 40 min at 46 °C, followed by enzyme inactivation for 1 min at 95 °C. The resulting cDNA was then stored at −20 °C for subsequent qPCR analysis [35].

### 4.9. Primer Design and Target Genes

Gene-specific primers (Table S5) were designed to amplify tyrosine hydroxylase (TH) in PC-12 cells and tryptophan hydroxylase (TPH) in Neuro-2A cells. The housekeeping genes β-actin and cyclophilin were used as internal reference controls for PC-12 and Neuro-2A cells, respectively, to normalize gene expression levels and control for variability between samples.

### 4.10. PCR Analysis

Quantitative PCR was conducted using SYBR Green Master Mix (Bio-Rad). Each reaction mixture contained cDNA, 10 µM of each forward and reverse primer, DNase-free water, and SYBR Green Supermix, prepared according to the manufacturer’s guidelines. Reactions were assembled in 384-well plates, with each experimental condition analyzed in triplicate to ensure statistical robustness. The plates were subjected to qPCR cycling using the following protocol by initial denaturation and followed by 40 amplification cycles. The relative expression levels of the target genes were determined using the ΔΔCt method, comparing treated samples to controls and normalizing against the corresponding reference gene [35].

### 4.11. Statistical Analysis

All data are presented as mean ± standard error of the mean (SEM). Statistical analysis of the results was conducted using one-way analysis of variance (ANOVA) in GraphPad Prism 10. The data were imported into GraphPad Prism 10 for both statistical analysis and graphical visualization. *p*-values of ≤0.05 were considered significant.

## 5. Conclusions and Future Perspectives

This study evaluated Hawaiian plant extracts for their neuroactive potential, identifying several promising candidates, A17 (*H. coronarium*), M19 (*P. albidus*), A40 (*C. ernodeoides*), M18 (*C. caesia*), and M30 (*M. citrifolia*) that significantly modulate TH and TPH gene expression. These enzymes play critical roles in dopamine and serotonin biosynthesis, suggesting that the active extracts may influence key neurotransmitter pathways implicated in neurodegenerative and mood disorders. The findings highlight the pharmacological richness of Hawaiian flora and validate the importance of ethnobotanical knowledge in guiding the discovery of novel bioactive compounds. Moreover, the differential effects observed among the extracts underscore the need for further phytochemical characterization to pinpoint the specific constituents that are responsible for their neuroactivity.

This study focused exclusively on aqueous and methanolic extracts, which are proficient in extracting polar to medium-polarity compounds such as polyphenols, flavonoids, glycosides, and polar alkaloids. While this approach successfully identified several active extracts, it inherently limits the scope of our findings. Important neuroactive non-polar constituents, including terpenoids, sterols, lipophilic alkaloids, and fatty acids, which are typically extracted with solvents like dichloromethane, ethyl acetate, or hexane, were not investigated. Consequently, the full neuropharmacological potential of the studied Hawaiian plants may not be fully captured. The activity we observed must therefore be interpreted as being mediated primarily by polar constituents, and future studies employing a sequential extraction protocol with solvents of varying polarity are essential to obtain a comprehensive phytochemical profile and bioactivity assessment.

A key limitation of the present study is its focus on transcriptional regulation via qPCR. While the upregulation of TH and TPH mRNA is a promising indicator of enhanced dopaminergic and serotonergic potential, it does not necessarily translate to a corresponding increase in functional enzyme protein or catalytic activity. Post-transcriptional regulation, protein stability, and post-translational modifications can all decouple mRNA levels from phenotypic outcomes. Therefore, our results, while significant, should be considered preliminary. To confirm a functional effect, future work must include Western blot analysis to quantify TH and TPH protein levels and enzymatic activity assays (e.g., via HPLC to measure the direct production of L-DOPA/dopamine or 5-HTP/serotonin) in treated cells. This will be crucial to establish a direct link between extract exposure and increased neurotransmitter synthesis.

It is critical to acknowledge that all experiments were conducted in an isolated in vitro cell culture system. This model does not account for the complex pharmacokinetic challenges a compound would face in vivo, including gastrointestinal degradation upon oral administration, absorption, plasma protein binding, systemic metabolism, and most importantly, blood–brain barrier penetration. An extract that is highly active in vitro may have little to no bioavailability or may be metabolized into inactive (or toxic) compounds in vivo. Thus, the potential suggested by our in vitro data remains speculative until these critical pharmacological parameters are evaluated in appropriate animal models.

Based on the limitations of the current work, the most active extracts will be subjected to bioassay-guided fractionation and detailed chemical analysis using Liquid Chromatography–High-Resolution Tandem Mass Spectrometry (LC-HR-MS/MS) to identify the specific bioactive compounds responsible for the observed gene modulation. Subsequent studies will move beyond gene expression to confirm functional activity through the quantification of TH and TPH protein levels (by Western blot) and their enzymatic products (by HPLC). The lead compounds or standardized fractions will be evaluated in appropriate rodent models to assess their bioavailability, metabolic stability, ability to cross the blood–brain barrier, and ultimately, their efficacy in promoting neuroprotection or influencing behavior related to dopaminergic and serotonergic pathways.

Future research will focus on bioassay-guided fractionation to isolate and structurally elucidate the active compounds, with particular emphasis on flavonoids, iridoids, and other secondary metabolites known for their neuroprotective and neuromodulators properties. Concurrently, mechanistic studies will investigate intracellular signaling pathways that regulate TH and TPH expressions, providing deeper insights into their molecular targets. Additionally, in vivo validation using preclinical models of Parkinson’s disease, depression, and related disorders will be essential to confirm efficacy, pharmacokinetics, and safety profiles. Advanced analytical techniques, including comparative metabolomics and transcriptomics, will further aid in identifying bioactive signatures and clarifying the mode of action of lead extracts, facilitating the development of standardized, evidence-based botanical formulations.

Beyond pharmacological applications, this research exemplifies a sustainable and integrative approach to drug discovery by bridging traditional ecological knowledge with modern science. The conservation of Hawaiian biodiversity and the preservation of indigenous medicinal practices are equally important outcomes of this work. By fostering collaborations between scientists, conservationists, and local communities, this initiative not only advances neuropharmacology but also promotes cultural heritage and environmental stewardship. Ultimately, the study lays a strong foundation for the development of next generation neurotherapeutics derived from nature, offering potential alternatives to synthetic drugs with fewer side effects and greater ecological compatibility.

## Data Availability

Data is contained within the article or Supplementary Materials.

## Supplementary Materials

The following supporting information can be downloaded at: https://www.mdpi.com/article/10.3390/ph18091403/s1, Figure S1: The percent cell viability on PC-12 treated with aqueous extracts of Hawaiian plants by the MTT assay; Figure S2: The percent cell viability on PC-12 treated with methanol extracts of Hawaiian plants by the MTT assay; Figure S3: The percent cell viability on Neuro-2A treated with aqueous extracts of Hawaiian plants by the MTT assay; Figure S4: The percent cell viability on Neuro-2A treated with methanol extracts of Hawaiian plants by the MTT assay; Table S1: List of the selected plants on PC-12 (Total 35 extracts); Table S2: List of the selected plants on Neuro-2A (Total 21 extracts); Table S3: Preliminary observations from the initial PCR of the selected plants on PC-12; Table S4: Preliminary observations from the initial PCR of the selected plants on Neuro-2A; Table S5: Primer sequences used in this study.

## Abbreviations

The following abbreviations are used in this manuscript:

cDNA	Complementary deoxyribonucleic acid
CREB	Cyclic adenosine monophosphate response element-binding protein
HPLC	High-Performance Liquid Chromatography
LC-HR-MS/MS	Liquid Chromatography–High-Resolution Tandem Mass Spectrometry
MAPK	Mitogen-activated protein kinase
MTT	3-(4, 5-dimethylthiazol-2-yl)-2,5-diphenyltetrazolium bromide
Nrf2	Nuclear factor erythroid 2-related factor 2
qPCR	Quantitative polymerase chain reaction
TH	Tyrosine hydroxylase
TPH	Tryptophan hydroxylase
