# Supplementary material for: Evaluation of Proliferative Activity of Hawaiian Plants on PC-12 and Neuro-2a Cells and Their Effect on the TPH and TH Genes
Source: Pharmaceuticals (Basel). 2025 Sep 18;18(9):1403. doi: 10.3390/ph18091403 (PMC12472671; doi:10.3390/ph18091403)
Supplement: Supplementary file 1 [file pharmaceuticals-18-01403-s001.zip › pharmaceuticals-3868380-supplementary.pdf]

Supplementary Materials

Evaluation of Proliferative Activity of Hawaiian Plants on PC-12 and Neuro-2a Cells and Their Effect on the TPH and TH Genes

Pornphimon Meesakul [1], Tyler Shea [2], Xiaohua Wu [1], Yutaka Kuroki [3], Aya Wada [3] and Shugeng Cao [1,*]

[1] Department of Pharmaceutical Sciences, Daniel K. Inouye College of Pharmacy, University of Hawai'i at Hilo, 200 W. Kawili St., Hilo, HI 96720, USA; pmeesak@hawaii.edu (P.M.); xiaohua3@hawaii.edu (X.W.)
[2] Chemistry Department, University of Hawai'i at Hilo, 200 W. Kawili St., Hilo, HI 96720, USA; tylerms3@hawaii.edu
[3] Delightex Pte. Ltd., 230 Victoria Street, #15-01/08 Bugis Junction Towers, Singapore 188024, Singapore; yutaka@delightexplorers.com (Y.K.); aya@delightexplorers.com (A.W.)
* Correspondence: scao@hawaii.edu

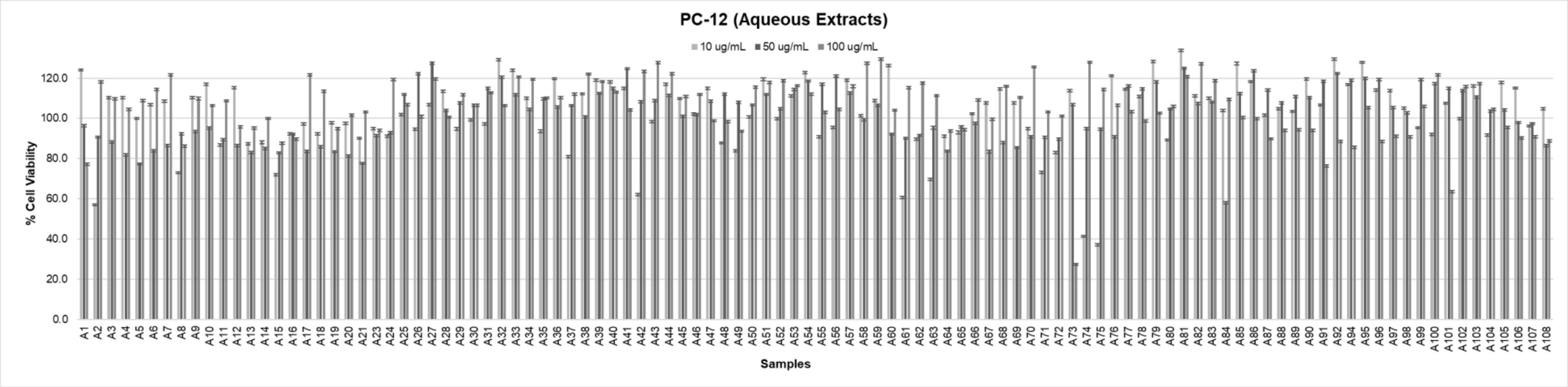

Figure S1. The percent cell viability on PC-12 treated with aqueous extracts of Hawaiian plants by the MTT assay. The data in the figure represents the mean ± SEM of triplicate experiments. $p < 0.05$ compared with the control group.

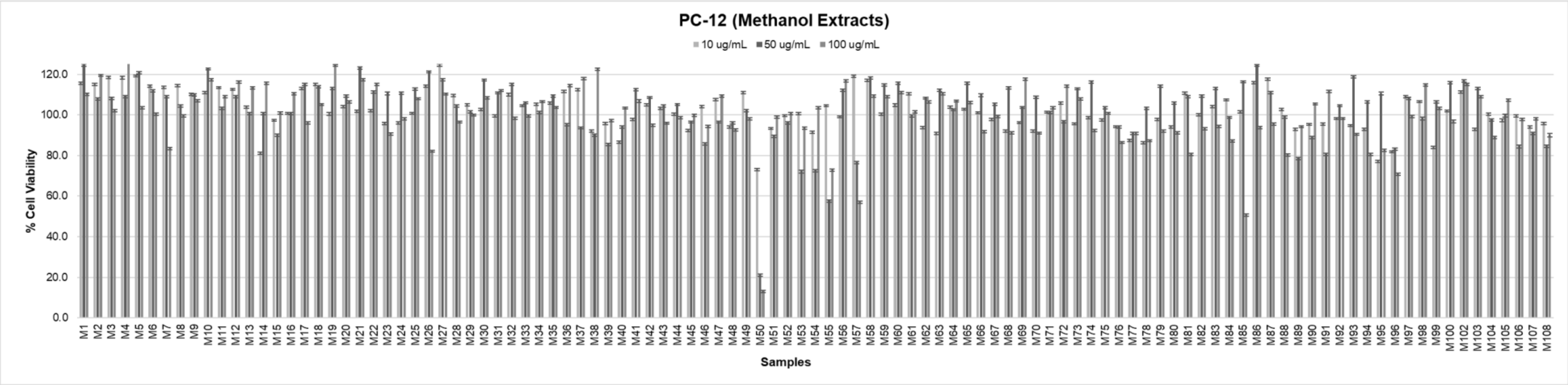

Figure S2. The percent cell viability on PC-12 treated with methanol extracts of Hawaiian plants by the MTT assay. The data in the figure represents the mean ± SEM of triplicate experiments. $p < 0.05$ compared with the control group.

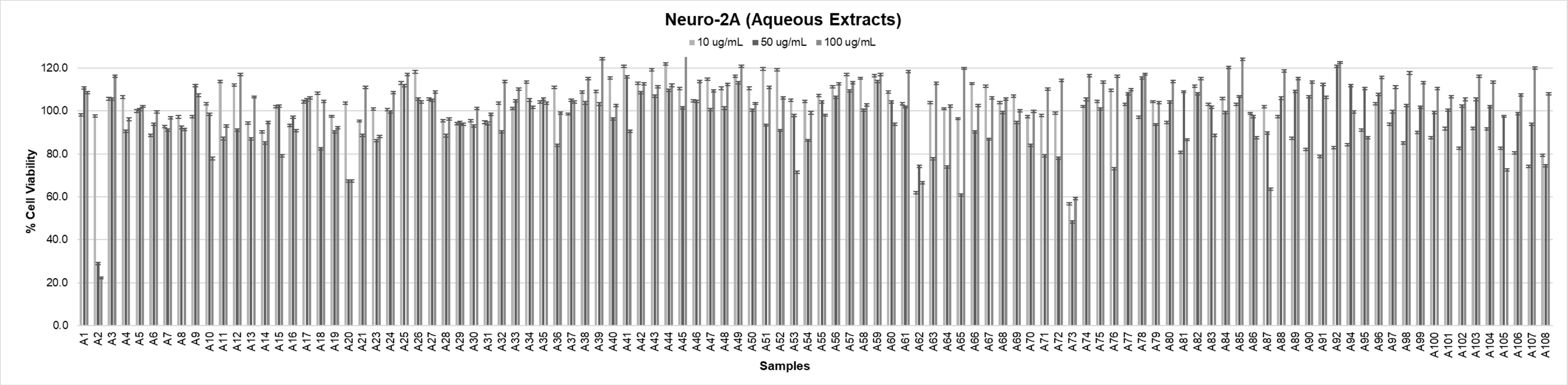

Figure S3. The percent cell viability on Neuro-2A treated with aqueous extracts of Hawaiian plants by the MTT assay. The data in the figure represents the mean ± SEM of triplicate experiments. $p < 0.05$ compared with the control group.

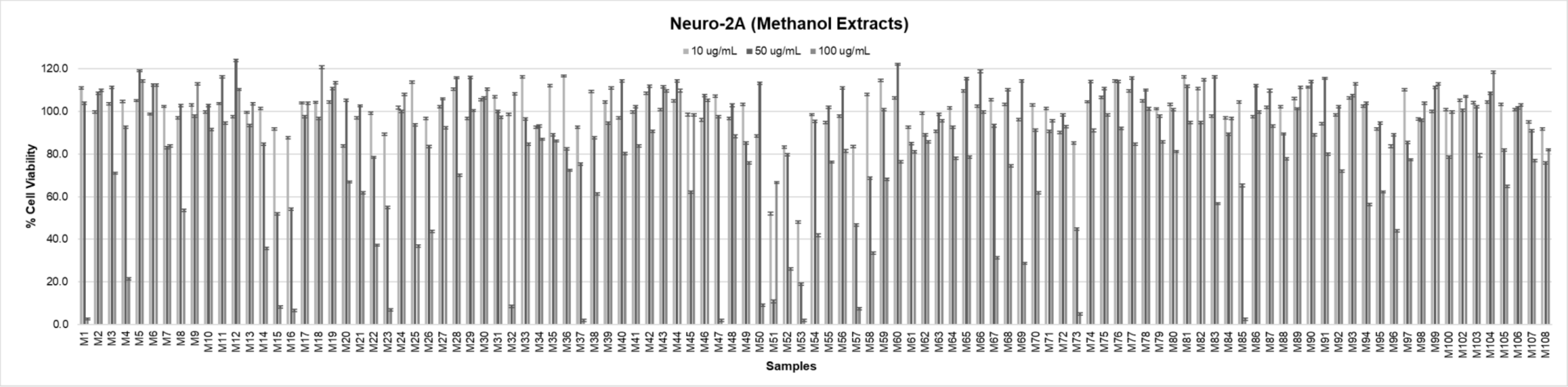

Figure S4. The percent cell viability on Neuro-2A treated with methanol extracts of Hawaiian plants by the MTT assay. The data in the figure represents the mean ± SEM of triplicate experiments. $p < 0.05$ compared with the control group.

Table S1. List of the selected plants on PC-12 (Total 35 extracts)

No.	Scientific Name	Status	Part	Sample Code	
				**Aqueous**[1]	**Methanol**[2]
1	*Verbascum thapsus*	Naturalized	Leaves	-	M3
2	*Curcuma longa*	Polynesia introduction	Rhizomes	-	M6
3	*Brunfelsia australis*	Naturalized	Leaves	-	M8
4	*Curcuma caesia*	Naturalized	Rhizomes	-	M18
5	Pipturus albudus	Endemic	leaves	-	M19
6	*Rubus hawaiensis*	Endemic	Stigs & leaves	-	M22
7	*Dicranopteris linearis*	Indigenous	Leaves	-	M27
8	*Podocarpus gracilior*	Naturalized	Leaves	A28	M28
9	*Hibiscus brackenridgei*	Endemic	Leaves, flowers	A29	M29
10	*Morinda citrifolia*	Polynesia introduction	Fruit	A30	M30
11	*Aleurites moluccana*	Naturalized	Leaves	-	M31
12	*Artocarpus altilis*	Polynesia introduction	Leaves	A32	-
13	*Coprosma ernodeoides*	Endemic	Leaves, twigs	A40	-
14	*Morinda citrifolia*	Polynesia introduction	Leaves	A43	-
15	*Myrsine lessertiana*	Endemic	Leaves	A47	-
16	*Cordyline fruticosa (green)*	Polynesia introduction	Leaves	-	M49
17	*Clusia rosea*	Naturalized	Fruit	A50	-
18	*Psilotum nudum*	Indigenous	Arial	A52	-
19	*Cordyline fruticosa (red)*	Polynesia introduction	Leaves	A53	-
20	*Crotalaria trichotoma*	Naturalized	Pods	A54	-
21	*Pipturus albudus*	Endemic	Twigs	-	M56
22	*Aleurites moluccanus*	Polynesia introduction	Fruirs	-	M69
23	*Buddleja asiatica*	Naturalized	Leaves	A78	-
24	*Cordia subcordata*	Indigenous	Leaves	A79	-
25	*Cordia subcordata*	Indigenous	Flowers	A81	-
26	*Argyreia speciosa*	Naturalized	Seeds	A95	-
27	*Argyreia speciosa*	Naturalized	Leaves/stems	-	M97
28	*Wikstroemia uva-ursi*	Endemic	Twigs	A99	-
29	*Cocos nucifera*	Polynesia introduction	Water	A101	-
30	*Pandanus tectorius*	Indigenous	Seed/fruit	A102	-
31	*Trema orientalis*	Naturalized	Leaves	-	M105
32	*Hippobroma longiflora*	Naturalized	Roots	A107	-

[1] A: Aqueous extracts, [2] M: Methanol extracts

Table S2. List of the selected plants on Neuro-2A (Total 21 extracts)

No.	Scientific Name	Status	Part	Sample Code	
				Aqueous[1]	Methanol[2]
1	*Annona muricata*	Naturalized	Leaves	-	M2
2	*Hedychium coronarium*	Naturalized	Rhizomes	A17	-
3	*Pipturus albudus*	Endemic	Leaves	-	M19
4	*Ipomoea batatas*	Polynesia introduction	Leaves	A26	-
5	*Morinda citrifolia*	Polynesia introduction	Fruit	-	M30
6	*Aleurites moluccana*	Naturalized	Leaves	-	M31
7	*Hibisccus tiliaceus*	Polynesia introduction	Leaves	A34	-
8	*Vaccinium reticulatum*	Endemic	Leaves, fruit	-	M42
9	*Crotalaria retusa*	Naturalized	Pods	A55	-
10	*Heterotis rotundifolia*	Naturalized	Whole plant	A74	-
11	*Buddleja asiatica*	Naturalized	Seeds	A77	-
12	*Buddleja asiatica*	Naturalized	Leaves	A78	-
13	*Buddleja asiatica*	Naturalized	Twigs	A80	-
14	*Cordia subcordata*	Indigenous	Flowers	-	M81
15	*Vitex rotundifolia*	Indigenous	Leaves	A85	-
16	*Prosopis pallida*	Naturalized	Seeds, pods	A88	-
17	*Rhodoria rosea*	Endemic	Roots	-	M93
18	*Chenopodium oahuense*	Endemic	Seeds	A96	-
19	*Wikstroemia uva-ursi*	Endemic	Twigs	-	M99
20	*Cocos nucifera*	Polynesia introduction	Endocarp	-	M104
21	*Trema orientalis*	Naturalized	Stems	-	M106

[1] A: Aqueous extracts, [2] M: Methanol extracts

Table S3. Preliminary observations from the initial PCR of the selected plants on PC-12.

No.	Scientific Name	Status	Part	Sample Code (Aqueous[1])	Expression Ratio ($2^{-\Delta\Delta Ct}$)	Sample Code (Methanol[2])	Expression Ratio ($2^{-\Delta\Delta Ct}$)
1	*Verbascum thapsus*	Naturalized	Leaves	-	-	M3	1.61
2	*Curcuma longa*	Polynesia introduction	Rhizomes	-	-	M6	1.70
3	*Brunfelsia australis*	Naturalized	Leaves	-	-	M8	1.15
4	*Curcuma caesia*	Naturalized	Rhizomes	-	-	M18	2.06
5	*Pipturus albudus*	Endemic	leaves	-	-	M19	0.23
6	*Rubus hawaiensis*	Endemic	Stigs & leaves	-	-	M22	0.62
7	*Dicranopteris linearis*	Indigenous	Leaves	-	-	M27	1.80
8	*Podocarpus gracilior*	Naturalized	Leaves	A28	1.18	M28	1.00
9	*Hibiscus brackenridgei*	Endemic	Leaves, flowers	A29	1.48	M29	0.62
10	*Morinda citrifolia*	Polynesia introduction	Fruit	A30	1.31	M30	2.04
11	*Aleurites moluccana*	Naturalized	Leaves	-	-	M31	0.79
12	*Artocarpus altilis*	Polynesia introduction	Leaves	A32	0.82	-	-
13	*Coprosma ernodeoides*	Endemic	Leaves, twigs	A40	2.66	-	-
14	*Morinda citrifolia*	Polynesia introduction	Leaves	A43	0.23	-	-
15	*Myrsine lessertiana*	Endemic	Leaves	A47	0.45	-	-
16	*Cordyline fruticosa (green)*	Polynesia introduction	Leaves	-	-	M49	0.21
17	*Clusia rosea*	Naturalized	Fruit	A50	0.38	-	-
18	*Psilotum nudum*	Indigenous	Arial	A52	0.45	-	-
19	*Cordyline fruticosa (red)*	Polynesia introduction	Leaves	A53	0.24	-	-
20	*Crotalaria trichotoma*	Naturalized	Pods	A54	0.36	-	-
21	*Pipturus albudus*	Endemic	Twigs	-	-	M56	0.77
22	*Aleurites moluccanus*	Polynesia introduction	Fruirs	-	-	M69	1.14
23	*Buddleja asiatica*	Naturalized	Leaves	A78	0.39	-	-
24	*Cordia subcordata*	Indigenous	Leaves	A79	0.48	-	-
25	*Cordia subcordata*	Indigenous	Flowers	A81	0.68	-	-
26	*Argyreia speciosa*	Naturalized	Seeds	A95	0.53	-	-
27	*Argyreia speciosa*	Naturalized	Leaves/stems	-		M97	0.59
28	*Wikstroemia uva-ursi*	Endemic	Twigs	A99	0.75	-	-
29	*Cocos nucifera*	Polynesia introduction	Water	A101	1.06	-	-
30	*Pandanus tectorius*	Indigenous	Seed/fruit	A102	1.26	-	-
31	*Trema orientalis*	Naturalized	Leaves	-	-	M105	1.27
32	*Hippobroma longiflora*	Naturalized	Roots	A107	1.70	-	-

[1] A: Aqueous extracts, [2] M: Methanol extracts

Table S4. Preliminary observations from the initial PCR of the selected plants on Neuro-2A.

No.	Scientific Name	Status	Part	Sample Code (Aqueous[1])	Expression Ratio ($2^{-\Delta\Delta Ct}$)	Sample Code (Methanol[2])	Expression Ratio ($2^{-\Delta\Delta Ct}$)
1	*Annona muricata*	Naturalized	Leaves	-	-	M2	1.04
2	*Hedychium coronarium*	Naturalized	Rhizomes	A17	2.34	-	-
3	*Pipturus albudus*	Endemic	Leaves	-	-	M19	2.42
4	*Ipomoea batatas*	Polynesia introduction	Leaves	A26	0.09	-	-
5	*Morinda citrifolia*	Polynesia introduction	Fruit	-	-	M30	1.89
6	*Aleurites moluccana*	Naturalized	Leaves	-	-	M31	0.76
7	*Hibisccus tiliaceus*	Polynesia introduction	Leaves	A34	0.13	-	-
8	*Vaccinium reticulatum*	Endemic	Leaves, fruit	-	-	M42	1.23
9	*Crotalaria retusa*	Naturalized	Pods	A55	0.24	-	-
10	*Heterotis rotundifolia*	Naturalized	Whole plant	A74	0.76	-	-
11	*Buddleja asiatica*	Naturalized	Seeds	A77	0.43	-	-
12	*Buddleja asiatica*	Naturalized	Leaves	A78	0.25	-	-
13	*Buddleja asiatica*	Naturalized	Twigs	A80	1.06	-	-
14	*Cordia subcordata*	Indigenous	Flowers	-	-	M81	1.30
15	*Vitex rotundifolia*	Indigenous	Leaves	A85	0.30	-	-
16	*Prosopis pallida*	Naturalized	Seeds, pods	A88	0.15	-	-
17	*Rhodoria rosea*	Endemic	Roots	-	-	M93	0.28
18	*Chenopodium oahuense*	Endemic	Seeds	A96	1.64	-	-
19	*Wikstroemia uva-ursi*	Endemic	Twigs	-	-	M99	1.66
20	*Cocos nucifera*	Polynesia introduction	Endocarp	-	-	M104	1.13
21	*Trema orientalis*	Naturalized	Stems	-	-	M106	0.55

[1] A: Aqueous extracts, [2] M: Methanol extracts

Table S5. Primer sequences used in this study.

Cell	Type of gene	Gene name	Direction	Sequence (5′–3′)
PC-12	Reference gene	Actin	Forward	AGGCCAACCGTGAAAAGATG
			Reverse	ACCAGAGGCATACAGGGACAA
	Target gene	TH	Forward	AGGGCTGCTGTCTTCCTAC
			Reverse	GCTGTGTCTGGGTCAAAGG
Neuro-2A	Reference gene	Cyclophilin	Forward	TCCGACTGTGGACAGCTCTA
			Reverse	ATTGCGAGCAGATGGGGTAG
	Target gene	TPH2	Forward	GCAAGACAGCGGTAGTGTTCT
			Reverse	CAGTCCACGAAGATTTCGACTT
